# Resilience dynamics and productivity‐driven shifts in the marine communities of the Western Mediterranean Sea

**DOI:** 10.1111/1365-2656.13648

**Published:** 2021-12-14

**Authors:** Manuel Hidalgo, Paraskevas Vasilakopoulos, Cristina García‐Ruiz, Antonio Esteban, Lucía López‐López, Elisa García‐Gorriz

**Affiliations:** ^1^ Instituto Español de Oceanografía (IEO, CSIC) Centro Oceanográfico de Baleares (COB) Ecosystem Oceanography Group (GRECO) Palma Balearic Islands Spain; ^2^ European Commission Joint Research Centre (JRC) Ispra Italy; ^3^ Instituto Español de Oceanografía (IEO, CSIC) Centro Oceanográfico de Málaga Fuengirola Málaga Spain; ^4^ Instituto Español de Oceanografía (IEO, CSIC) Centro Oceanográfico de Murcia San Pedro del Pinar Murcia Spain

**Keywords:** benthopelagic communities, critical transitions, life‐history strategies, Mediterranean Sea, resilience dynamics

## Abstract

1. Ecological resilience has become a conceptual cornerstone bridging ecological processes to conservation needs. Global change is increasingly associated with local changes in environmental conditions that can cause abrupt ecosystem reorganizations attending to system‐specific resilience fluctuations with time (i.e. resilience dynamics).
2. Here we assess resilience dynamics associated with climate‐driven ecosystems transitions, expressed as changes in the relevant contribution of species with different life‐history strategies, in two benthopelagic systems.
3. We analysed data from 1994 to 2019 coming from a scientific bottom trawl survey in two environmentally contrasting ecosystems in the Western Mediterranean Sea—Northern Spain and Alboran Sea. Benthopelagic species were categorized according to their life‐history strategies (opportunistic, periodic and equilibrium), ecosystem functions and habitats. We implemented an Integrated Resilience Assessment (IRA) to elucidate the response mechanism of the studied ecosystems to several candidate environmental stressors and quantify the ecosystems’ resilience. We demonstrate that both ecosystems responded discontinuously to changes in chlorophyll‐*a* concentration more than any other stressor. The response in Northern Spain indicated a more overarching regime shift than in the Alboran Sea. Opportunistic fish were unfavoured in both ecosystems in the recent periods, while invertebrate species of short life cycle were generally favoured, particularly benthic species in the Alboran Sea.
4. The study illustrates that the resilience dynamics of the two ecosystems were mostly associated with fluctuating productivity, but subtle and long‐term effects from sea warming and fishing reduction were also discernible. Such dynamics are typical of systems with wide environmental gradient such as the Northern Spain, as well as systems with highly hydrodynamic and of biogeographical complexity such as the Alboran Sea. We stress that management should become more adaptive by utilizing the knowledge on the systems’ productivity thresholds and underlying shifts to help anticipate both short‐term/less predictable events and long‐term/expected effects of climate change.

## Graphical abstract

The study demonstrates that similar primary productivity shocks in the Western Mediterranean triggered benthopelagic community shifts in neighbouring ecosystems exhibiting contrasting resilience dynamics and contribution of species with different life‐history strategies.

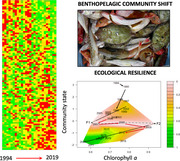

## 1 INTRODUCTION

Resilience has become a conceptual cornerstone in marine management and a popular narrative for marine conservation as it provides the opportunity to communicate ecosystem health and the capacity to overcome both natural and anthropogenic disturbances (Darling & Côté, 2018). However, its applications and implications are still partially disconnected from the ecological basis (Johnson & Lidström, 2018). This is largely due to the difficulty to find metrics and methods to apply efficiently the resilience theory in an empirical context (e.g. Collie et al., 2004; Scheffer, 2009 but see, Vasilakopoulos et al., 2017; Sguotti et al., 2019; Capdevila et al., 2020).

Resilience is generally defined in two complementary ways in ecological literature: ecological and engineering resilience. Ecological resilience is the capacity of a system to absorb disturbance and reorganize while undergoing change so as to retain essentially the same function, structure, identity and feedbacks (Walker et al., 2004). By contrast, engineering resilience is an expression of the time required for an ecosystem to return to an equilibrium or steady state following a perturbation (Holling, 1996). While engineering resilience focuses on the stability around a single equilibrium, ecological resilience emphasizes conditions far from the equilibrium, where a system may flip to an alternate domain (Holling, 1996). In any case, both the ability of ecosystems to resist and absorb disturbance without changing basin of attraction (ecological resilience) and their rate of recovery towards a prevailing steady state (engineering resilience) are complementary emergent properties to be secured for the oceans’ sustainability.

Herein, ‘resilience’ refers to ecological resilience, unless otherwise specified, as it is based on the theory of critical transitions and alternate stable states (Scheffer, 2009). Complex natural systems with eroded resilience, ranging from populations to ecosystems and socioecological systems, can exhibit abrupt reorganizations in their components in response to external stressors (e.g. Litzow & Hunsicker, 2016; Scheffer, 2009; Scheffer et al., 2001). These system reorganizations are broadly known as regime shifts, defined as large and abrupt changes in the structure and function of complex systems. Different types of shifts can be identified attending to the relationship between the ecosystem state(s) and the fluctuations of the driver(s), broadly categorized as continuous or discontinuous (Scheffer et al., 2001; Selkoe et al., 2015).

Regime shifts alter the properties of ecosystems by triggering persistent changes in their structure, dynamics and internal feedbacks, which can affect specific ecosystem functions (Möllmann et al., 2015). To operationalize resilience in ecosystem‐based management, it is crucial to identify which functions, and associated species, are (un)favoured in alternate regimes. Recent studies have suggested, for instance, that pelagic and thermophilic species of smaller size and of low trophic level will benefit from sea warming scenarios in temperate ecosystems (e.g. Moullec et al., 2019). While large‐scale thermal gradients shape geographical variation in marine traits (Pecuchet et al., 2017), regional variation is adapted to local environmental variability. Identifying which traits and functions are favoured in the alternative stable states before and after a regime shift would allow to identify specific management measures tailored to the new ecosystem and environmental realities at regional scale. This may help to avoid chronic and profound impacts that could hamper ecosystem services, particularly in environmentally heterogeneous systems.

Beyond ocean warming, climate change impacts are also associated with less predictable changes in environmental conditions, as well as with extreme weather events such as heatwaves or strong winter storms. While extreme events are mainly known to cause mass mortality episodes, they can also cause a reorganization of the ecosystem components (Maxwell et al., 2019). However, in ecosystems with low resilience, small changes in the environmental conditions can also trigger drastic changes in the prevailing regime with substantial modification in key functions and the most frequent traits in the communities. Such environmental drivers include also changes in primary production (i.e. productivity shifts) that can cascade fast in the food web and trigger strong modifications on the upper trophic levels (Fu et al., 2018).

The Mediterranean Sea is also one of the most exposed areas worldwide to the numerous impacts of climate change, including one of the highest warming rates, and increasing frequency of extreme weather events, and also subjected to productivity changes (Macias et al., 2018). It is also exposed to multiple anthropogenic impacts, such as overfishing and invasive species (e.g. Coll et al., 2012). The cumulative effects of different impacts and the patched structure of marine populations and communities make Mediterranean systems highly responsive to environmental fluctuations, and their resilient capacities highly driven by climate variability including primary production changes (Piroddi et al., 2017).

Here, we hypothesize that putative recent climate‐driven shifts are occurring in the Western Mediterranean associated with changes in the relative contribution of different groups of species with different life‐history strategies and ecosystem functions. To assess this hypothesis, we investigate the temporal development of two environmentally contrasting benthopelagic ecosystems of the Western Mediterranean Sea during 1994–2019, using data coming from scientific bottom trawl surveys. We first examine both the multivariate development of the ecosystems as a whole and the relative contribution of groups of species with different life‐history strategies and ecosystem functions, to understand the observed reorganizations. We then implement an Integrated Resilience Assessment (IRA; Vasilakopoulos & Marshall, 2015; Vasilakopoulos et al., 2017) to assess the response type of the ecosystems against several candidate environmental stressors, quantify ecological resilience and its temporal variation (i.e. resilience dynamics), and build stability landscapes with alternate valleys of attraction.

## 2 MATERIALS AND METHODS

### 2.1 Systems and data

**FIGURE 1:**
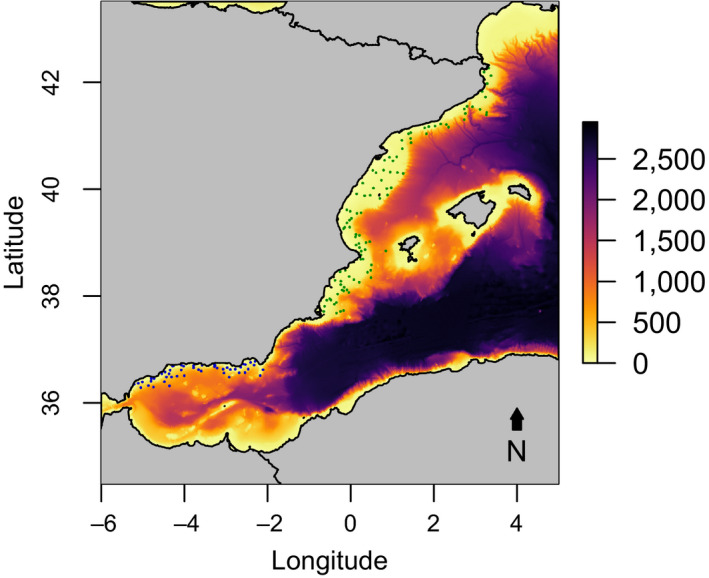
Distribution of the stations sampled by the MEDITS program in Northern Spain (green dots) and in Alboran (blue dots). Bar scale refers to the bathymetry (in meters)

We focus on two environmentally contrasting demersal ecosystems: in Northern Spain and in the Northern Alboran Sea (hereafter referred as ‘Alboran’; Figure 1). The Northern Spain system corresponds to the eastern Balearic Sea associated with the Iberian Peninsula coast and is characterized by large‐scale environmental gradients, including surface and intermediate water masses temperature, and primary productivity regimes resulting from the confluence of deep ocean convection from the Gulf of Lions, riverine nutrient inputs by the Ebro river and sub‐mesoscale and frontal processes (Ramirez‐Romero et al., 2020). The Alboran is, by contrast, a transitional system between the Atlantic and the Mediterranean ecosystems with strong hydrodynamism characterized by two permanent anticyclonic gyres encircled by the strong eastward flow and a higher mean primary productivity compared to Northern Spain.

This study used information obtained from a scientific bottom trawl survey (MEDITS program; note that ethical approval was not required for this research), conducted to evaluate the status of demersal resources in the early summer (May–June) of every year from 1994 to 2019. Here, we focused on the demersal and megaepibenthic community of the continental shelf (<200 m; Figure 1), having sampled 1,436 and 391 hauls in the Northern Spain and the Alboran, respectively, with an average depth of 89 m and 87 m. Data used in this study were retrieved at species level, averaging biomass per swept area (i.e. kg/km^2^) caught for each species and sampling station in every year.

With regards to environmental data, we focused on three potential drivers of the biological system: chlorophyll concentration (Chl *a*), sea surface temperature (SST) and an integrated Regional Hydroclimatic Index (RHI). These variables were selected attending to observed species‐specific responses and low trophic level effects in previous studies in the same areas, in both pelagic and demersal ecosystems (e.g. Puerta et al., 2014). We retrieved seasonal records of environmental variables to calculate two seasonal averages: for spring (March–May) and winter (December–February), assuming that species more sensitive to changes in primary production may be more associated with the winter signal as it is associated with the main phytoplankton bloom. SST information at 1/16 degree resolution for 1992–2019 was obtained from the Mediterranean physics re‐analyses available at the Copernicus Marine Service (https://marine.copernicus.eu).

To calculate a Regional Hydroclimatic Index (RHI), several environmental variables were obtained from the NCEP‐DOE Reanalysis 2 fields provided by NOAA (https://psl.noaa.gov/data/gridded/data.ncep.reanalysis2.html) for 1992–2019. Anomaly fields were retrieved at a monthly scale for air temperature, sea surface temperature, atmospheric sea level pressure, 500 hPa geopotential height and precipitation rates. The RHI quantifies an integrated hydroclimatic variability at regional scale synthesizing the aforementioned variables by means of the first axis of a Principal Component Analyses (PCA). High RHI values are associated with higher atmospheric sea level pressure and 500 hPa geopotential height, and negative values with high precipitation rate, both in Northern Spain and in Alboran.

Since satellite observations of Chl *a* from the Copernicus Marine Service were only available from 1998 onwards, Chl *a* information for 1992–2019 was obtained using the Mediterranean Ecological Regional Ocean Model (the MedERGOM biogeochemical model) coupled to the General Estuarine Transport Model (the GETM 3D hydrodynamic model), as described in Macias et al. (2019). These MedERGOM/GETM results have a horizontal resolution of 5′ × 5′ (~9 × 9 km). The implementation for the Mediterranean Sea is forced at surface every 6 hr by atmospheric data from the European Centre for Medium‐Range Weather Forecasts—ECMWF (Macias et al., 2019). Comparison of the computed Chl *a* estimates with high‐resolution observational datasets shows a high consistency at spatial and interannual levels, but some discrepancy at monthly scale related to the performance of Ocean Colour Chl *a* retrieval algorithm and intrinsic model errors not affecting the interannual pattern (Macias et al., 2019).

### 2.2 Integrated Resilience Assessment

The IRA is a three‐step methodological framework which applies the concepts of resilience and folded stability landscapes in an empirical multivariate context through the combination of multivariate analysis, non‐additive modelling and a resilience assessment (Vasilakopoulos et al., 2017). This way, the IRA elucidates the system dynamics and shift mechanisms in response to external stressors.

As a first step, a PCA was applied to the time‐species matrix of each studied area to identify the main modes of community variability. The PCAs were based on the correlation matrices of the species’ biomasses, following log‐transformation. PCA converts a set of possibly correlated variables into new linearly uncorrelated composite variables (principal components—PCs) using an orthogonal transformation of the data to a new coordinate system (Vasilakopoulos & Marshall, 2015). The standardized value that each data point has in the PC coordinate system (PC‐score) is then calculated. As the majority of individual species reported in the two ecosystems in 1994–2019 were caught only sporadically, we applied the PCA on six datasets for each area, including species caught in all 26 years or at least in 25, 24, 23, 22 or 21 years, respectively. This way, it was examined how the perception of the system dynamics would change in response to the number of species analysed while ensuring that only well‐sampled species would be retained. That ‘optimal’ PCA selected was the most parsimonious one (i.e. with the lowest number of species), as long as it captured similar dynamics with the ones including more species.

The sequential regime shift detection method (STARS), modified to account for temporal autocorrelation (Rodionov, 2006), was applied to detect significant shifts in the mean values of PC1 and PC2 of the optimal PCAs. STARS estimates a Regime Shift Index (RSI), that is, a cumulative sum of normalized anomalies relative to each value of the time series analysed, and uses it to test the hypothesis of a regime shift occurring in that year (Rodionov, 2006). Here, we used a cut‐off length of 3 years and a significant probability threshold of *p* = 0.05.

The composite variable (PC1 or PC2) retained to be further analysed within the IRA was selected based on its capacity to capture directional changes and non‐overlapping states, and on the degree of its coupling with the environmental variables. To identify the key species driving the community trends through time, the contributions of all species on PC‐scores (loadings) of the retained PCs were checked.

To investigate the type of the relationship between the biological system, as captured by PC1 or PC2, and each of the environmental stressors (winter and spring Chl *a*, SST and RHI), the fits of relevant generalized additive models (GAMs) and non‐additive threshold GAMs (TGAMs) at 0‐ to 2‐time lags were compared. The effect of the stressors on the system was examined at 0‐ to 2‐year lags to account for a potential delay in the environmental effect on the community sampled given that environmental effects typically influence spawning and early life stages, while the sampled biomass is usually dominated by specimens older than 0 years old. This maximum of 2 years is in line with previous Mediterranean studies (Damalas et al., 2021; Tsimara et al., 2021; Vasilakopoulos et al., 2017), and it was selected because of the narrow demography of Mediterranean fish species (Colloca et al., 2013) and their generally low generation time. For fish, *Merluccius merluccius* was the species with the highest generation time and above 2 years, with the rest of the species likely below this value (expect elasmobranchs). Crustaceans and cephalopods have life cycles of 2–3 years and 1 year, respectively.

Generalized additive models assume additive and stationary relationships between the response and explanatory variables, while TGAMs can represent an abrupt change in the relationships between the response and explanatory variables (i.e. a threshold) in a specific year supporting the occurrence of a regime shift (Ciannelli et al., 2004). In other words, a GAM describes a system that changes in a continuous way in response to the corresponding change of its stressor(s), while a TGAM represents a system response curve that is folded backwards, forming a fold bifurcation with two tipping points (Figure S1). Accordingly, we fit 18 GAMs and 18 TGAMs (3 stressors × 2 seasons × 3 lags) for each system, using either PC1 or PC2 as a response variable. To compare the goodness of fit of GAMs and TGAMs and select the optimal model, we computed the ‘genuine’ cross‐validation squared prediction error (genuine CV; gCV), which accounts for the estimation of the threshold line and the estimation of the degrees of freedom for the functions appearing in all additive and non‐additive formulations (Ciannelli et al., 2004). Additionally, a sensitivity analysis was carried out using also PC3 as response variable for the GAM‐TGAM comparison, to confirm that PC1 or PC2 captured best the system response to environmental stressors in both systems.

In both Northern Spain and Alboran, the branches of the respective optimal TGAMs identified earlier (i.e. the ones with the lowest gCV values) were considered as the attractors forming the fold bifurcations, in accordance with the theory of critical transitions (Scheffer et al., 2001). Subsequently, the resilience of each annual system state within the fold‐bifurcation was quantified based on the distance of each state from its attractor and tipping point (Vasilakopoulos & Marshall, 2015). Specifically, to estimate annual resilience values (*Res*_y_), the sum of the horizontal distance of each annual system state from the tipping point of its regime minus the vertical distance of each annual system state from its fitted attractor (TGAM branch) was used (Figure S1). This calculation requires the *x*‐ and the *y*‐axes of the bifurcation diagram to be measured at the same scale; hence, both the environmental stressor and the system PC were standardized (mean of 0 and standard deviation of 1; Vasilakopoulos et al., 2017). To calculate the position of the tipping point of each regime along the trajectory of its respective attractor, the *x*‐coordinates of the tipping points were set so as to ensure that the lowest *Res*_y_ estimate within each regime was equal to zero. Finally, *Res*_y_ was scaled by dividing with the maximum value observed to calculate relative resilience (*rRes*_y_). The stability landscape with its alternate basins of attraction emerged through linear interpolation of all *rRes*_y_ values onto a 100 × 100 grid.

The IRA was carried out in R (R Core Team, 2019) using packages vegan (Oksanen et al., 2019), mgcv (Wood, 2017) and akima (Akima et al., 2015).

### 2.3 Species indicators relevant to taxonomic, functional and temperature affinity

To quantify which species groups were favoured (‘winners’) and unfavoured (‘losers’) during 1994–2019 with regards to their taxonomy, habitat, life‐history strategies and temperature preferences, we calculated a range of different biomass indicators using the same set of species as in the optimal PCAs described in Section 1, 2.1.

For fish, we assessed the temporal development of the functional community composition according to three life‐history strategies—opportunistic, periodic and equilibrium—resulting from trade‐offs between traits (Pecuchet et al., 2017; Winemiller & Rose, 1992). The opportunistic strategy refers to species with small size, low trophic level and short life span but with relatively high fecundity and low generation time; for instance, species of the family Gobiidae and small pelagic fish. This strategy is favoured in environmental settings dominated by unpredictable and highly dynamic environmental variation (Winemiller & Rose, 1992). The periodic strategy refers to species with medium to long life span and length, high trophic level and high fecundity, but low parental care and offspring size; this is the case of conger eel *Conger conger* and species of gadoids such *Merluccius merluccius* and *Phycis blennoides*, and also monkfish species (*Lophius* spp). The equilibrium strategy characterizes species with long length and life span and high trophic level, low fecundity but large offspring size and high parental care, such as most elasmobranchs. This strategy is favoured in more stable, less impacted and more predictable environments. Pecuchet et al. (2017) quantified the relative contribution of each strategy for each species analysing fish communities of the whole Europe and applying an archetypal analysis on the life‐history characteristics of all fish species reflecting reproductive, growth and feeding modes. This approach is based on the identification of points (archetypes) forming the corners of the convex hull volume encompassing the trait space; points are then represented by proportions based on the proximity of each point to each archetype (more details in Pecuchet et al., 2017). For this study, we used the relative value associated with each life‐history strategy, with the three values summing 1. Log‐transformed biomass of each species was used to calculate a weighted mean of the values associated with each life‐history strategy. Fish biomass was also calculated in terms of commercial and non‐commercial species.

For crustaceans and cephalopods, we calculated the mean biomass for the two main habitats: ‘benthic’, where the animals are comparatively more impacted by fishing activities, and pelagic/benthopelagic (hereafter referred as ‘pelagic’) being relatively more influenced by environmental variability. Prior to the calculations, to give an equal weight to all species and avoid a higher influence of species with higher mean biomass, log‐transformed biomass of each species was standardized (mean of 0 and standard deviation of 1).

To evaluate the potential increase in species with higher affinity to warmer temperatures, we used the information of the *AQUAMAPS* database (https://www.aquamaps.org) to extract the optimal temperature of the habitat distribution for each species, using only the area of distribution of species with probability of occurrence higher than 50% and weighing the temperature by the probability of occurrence. For the few species for which the information was not available in *AQUAMAPS*, we calculated their preferred temperature based on the mean value of the presence data recorded in the Ocean Biodiversity Information Systems (OBIS, https://obis.org). Finally, we calculated the community‐weighted mean temperature (CWMT; Cheung et al., 2013) for each year as ${\text{CWMT}}_{i}=\sum Tpref_{s}*W_{s,i}/\sum W_{s,i}$, where *Tpref_s_* is the preferred temperature of a species *s* and *W_s,i_* is the log‐transformed biomass in weight of species *s* in the year *i*.

All the time series of life‐history contributions, taxonomic groups and CWMT were explored in relation to the putative regime shifts identified. In addition, temporal trends were statistically tested in all the species’ indicators fitting linear models using generalized least squares (function *gls* of *nlms* library in R), in which the errors are allowed to be correlated attending to their level of autocorrelation structure. In most cases, this structure takes the form of an autoregressive process of order 1 (i.e. corAR1)—that is, an autoregression mode that forecasts the model errors using a linear combination of their past values, being lagged one year in the case of corAR1. Only those models with statistically significant trends were identified and presented in the relevant figures.

## 3 RESULTS

The total number of individual species caught by the survey in Northern Spain and Alboran in 1994–2019 was 483 and 415, respectively. However, the number of species encountered in all 26 years was 75 in Northern Spain and 38 in Alboran. The ‘optimal PCA’ selected was the one including species present in at least 25 years in Northern Spain (97 species; Figure S2) and the one including species present in at least 21 years in Alboran (74 species; Figure S3).

**FIGURE 2:**
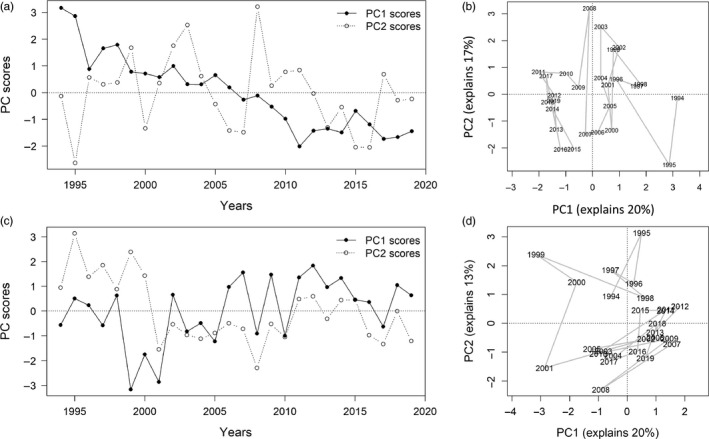
Temporal variation of the main modes of the community variability revealed by the yearly PC‐scores for the first two principal components of the PCAs applied for the Northern Spain (above) and Alboran (below). Time series of PC‐scores of the two modes (a, c) and their biplot (b, d)

**FIGURE 3:**
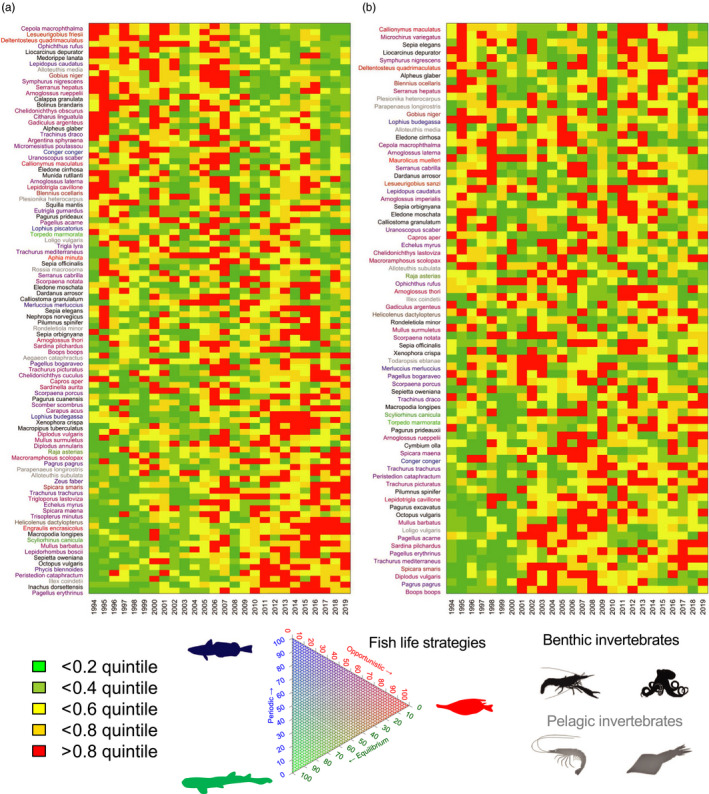
Changes in the ecosystem configuration (i.e. relative contribution of species) attending to the temporal development of standardized biomass of 97 species in Northern Spain sorted according to their loadings on PC1 (a) of 74 species in Alboran sorted according to their loadings on PC2 (b). Note PCs presented are those showing directional changes and non‐overlapping states, and thus selected as community indicators in the IRA

In both Northern Spain and Alboran, PC1 explained 20% of the total deviance, owing to the high number of species included in the respective PCAs. In Northern Spain, the community modes showed a clear monotonic trend in PC1, with the biplot of PC1 versus PC2 showing a transition along the PC1 axis in 1995–1996 and another one in the late 2000s (Figure 2a,b). These changes in the ecosystem configuration were well captured by the traffic light plot which indicated a large number of species increasing or decreasing progressively over time (Figure 3a). In Alboran, the community change was not as pronounced or encompassing as in Northern Spain. PC1 did not exhibit a monotonic trend and was relatively stationary over the whole period (Figure 2c), while sorting species according to their loadings on PC1 in a traffic light plot suggested a limited directional change in the community (Figure S4). Instead, a transition in 2000–2001 was observed in the PC2. This pattern in PC2 coincided with a concurrent change in a group of species that decreased after 2000 and rebounded in the early 2010s, and another group of species that increased sharply after 2000 (Figure 3b). All these transition patterns were confirmed by STARS (Figure S5).

**FIGURE 4:**
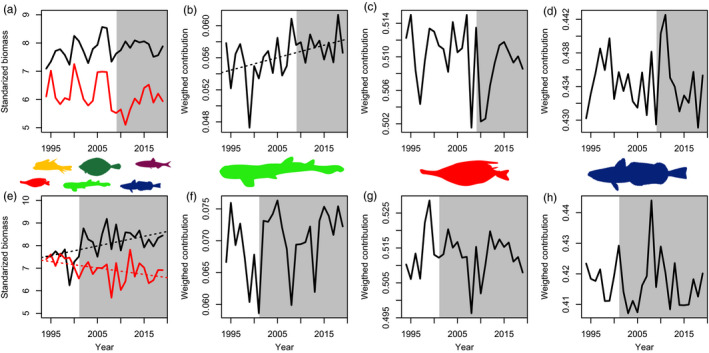
Fish species trends for Northern Spain (above) and Alboran (below): standardized biomass of commercial (black line) and non‐commercial species (red line; a, e), and weighted contribution for the three life‐history strategies, equilibrium (b, f), opportunistic (c, g) and periodic (d, h). Dotted black lines indicate that the temporal trends are significant over the whole period (*p* < 0.05; only statistically significant temporal trends are presented). Grey background represents the new ecosystems states after the regime shifts identified, that is, from 2009 and 2001 in Northern Spain and Alboran, respectively

**FIGURE 5:**
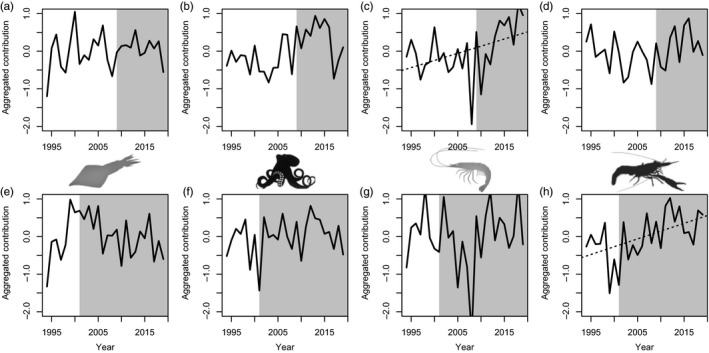
Averaged contribution of cephalopods and crustaceans associated with different habitats for Northern Spain (above) and Alboran (below): pelagic (a, e) and benthic (b, f) cephalopods, and pelagic (c, g) and benthic (d, h) crustaceans. Dotted black lines indicate that the temporal trend is significant over the whole period (*p* < 0.05; only statistically significant temporal trends are presented). Grey background represents the new ecosystems states after the regime shifts identified, that is, after 2009 and 2001 in Northern Spain and Alboran, respectively

**FIGURE 6:**
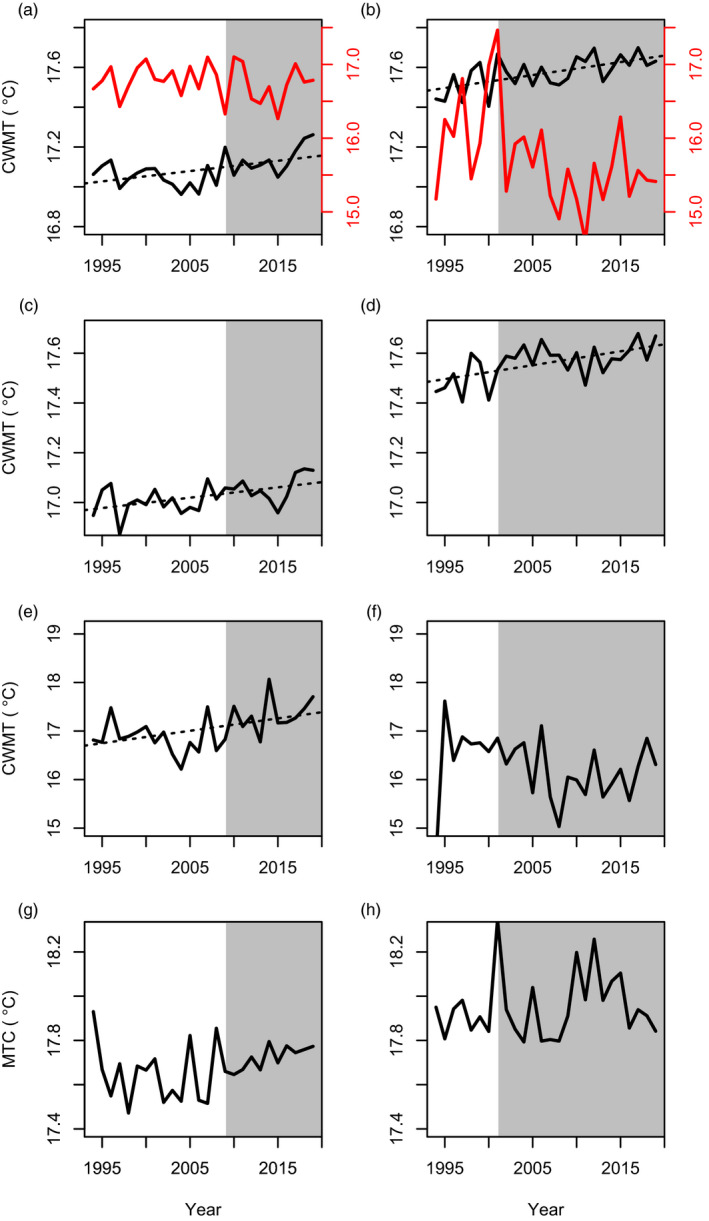
Community‐weighted mean temperature (CWMT, °C) of the demersal community in Northern Spain (left) and Alboran (right): commercial (black line) and non‐commercial species in the whole community (a, b), fish (c, d), crustaceans (e, f) and cephalopods (g, h). Dotted black lines indicate that the temporal trend is significant over the whole period (*p* < 0.05; only statistically significant temporal trends are presented). Grey background represents the new ecosystem states after the regime shifts identified, that is, after 2009 and 2001 in Northern Spain and Alboran, respectively

The transitions identified in the late 2000s and early 2000s in the Northern Spain and Alboran, respectively, were associated with different groups of species, with different functions and life‐history strategies being favoured and unfavoured in each ecosystem. In Northern Spain, all taxonomic groups displayed clear changes associated with the late 2000s transition, with commercial and non‐commercial fish showing a relative synchronous pattern (Figure 4a). Certain fish species declined sharply, especially the opportunistic non‐commercial ones and a few periodic species (Figures 3a and 4; Table S1). By contrast, other fish species, particularly commercial ones were favoured during the community transition, while no obvious trend was observed in the opportunistic and periodic species (Figure 4). Equilibrium species (e.g. elasmobranchs) exhibited a significant increasing trend (slope(sl) = 0.0001, *p* < 0.05, Figure 4). For cephalopods and crustaceans (Figure 5), the late 2000s was also a critical period with extremely low values for species of pelagic behaviour, particularly evident in the case of pelagic crustaceans displaying a statistically significant trend (sl = 0.037, *p* < 0.05). The biomass of benthic cephalopods also increased after ca. 2007, with a progressive increase also observed in the benthic crustaceans (Figure 5). Regarding community‐weighted mean temperature (CWMT) of Northern Spain, a significant increase was observed in the whole time series for species of commercial interest (sl = 0.006, *p* < 0.05, Figure 6). This trend was consistently observed across all taxonomic groups, while only significant in fish and crustaceans (sl = 0.004 and sl = 0.025, respectively, both *p* < 0.05, Figure 6).

In Alboran, unlike in Northern Spain, significant long‐term decreasing and increasing trends were observed for non‐commercial and commercial species, respectively (sl = −0.02 and sl = 0.04, both *p* < 0.05), mainly referring to sparids and carangids (Figure 4e; Table S2). This is consistent with the community shift observed in the early 2000s that, in respect to fish, was partially related to a decrease in the contribution of opportunistic fish species (Figure 4g). Some less pronounced change was also observed in equilibrium and periodic fish species. The temporal development of crustaceans and cephalopods reflected the early 2000s shift with a general decrease in pelagic invertebrates (mainly cephalopods) and an increase in both benthic cephalopods and crustaceans (Figure 5), with a significant trend in the latter (sl = 0.004, both *p* < 0.01, Figure 5). The estimates of the CWMT also showed a general increase for commercial species over the study period (sl = 0.005, *p* < 0.05, Figure 6), while a sharp decrease associated with the early 2000s shift was observed in the CWMT of the non‐commercial species. The increase in CWMT was mainly associated with fish (sl = 0.005, *p* < 0.05, Figure 6). Non‐commercial CWMT decrease was associated with crustacean species (Figure 6f).

### 3.1 Integrated Resilience Assessment

PC1 and PC2 captured better than other PCs the directional underlying trends in Northern Spain and Alboran, respectively, and exhibited non‐overlapping regimes (Figure 2). Additionally, the examination of all continuous (GAMs) and discontinuous (TGAMs) fits using PC1 or PC2 as a response variable and all candidate stressors at 0‐ to 2‐year lags as explanatory variables, revealed that the a TGAM of PC1 with winter Chl *a* at a 2‐year lag and a TGAM of PC2 with winter Chl *a* at an 1‐year lag provided the best fits for Northern Spain and Alboran, respectively (lowest gCV; Table 1; Figure S6). The sensitivity analysis using PC3 as a response variable in both areas did not yield any stronger coupling with environmental stressors (Table 1). Hence, PC1 was retained as system indicator (PC1sys) for the next steps of the IRA in Northern Spain, while PC2 (PC2sys) was retained for Alboran.

**TABLE 1:** The optimal (lowest gCV) GAM or TGAM models identified using PC1, PC2 or PC3 as a response variable and Chl *a*, SST and RHI of spring and winter at 0‐ to 2‐year lags as explanatory variables in the Northern Spain and Alboran systems. In bold, the model exhibiting the best fit for each area. All PCs were standardized prior to the GAM‐TGAM fitting, thus making gCVs comparable

Response variable	Stressor	Type of model	Lag	gCV	Variance explained (%)
Northern Spain					
**PC1**	**Chl *a*_winter**	**TGAM**	**2 years**	**0.217**	**87.4**
PC2	Chl *a*_winter	GAM	1 year	0.927	24.9
PC3	Chl *a*_winter	TGAM	2 years	0.569	69.4
Alboran					
PC1	SST_winter	TGAM	2 years	0.716	57.3
**PC2**	**Chl *a_*winter**	**TGAM**	**1 year**	**0.423**	**74.1**
PC3	SST_winter	TGAM	2 years	0.587	68.3

**FIGURE 7:**
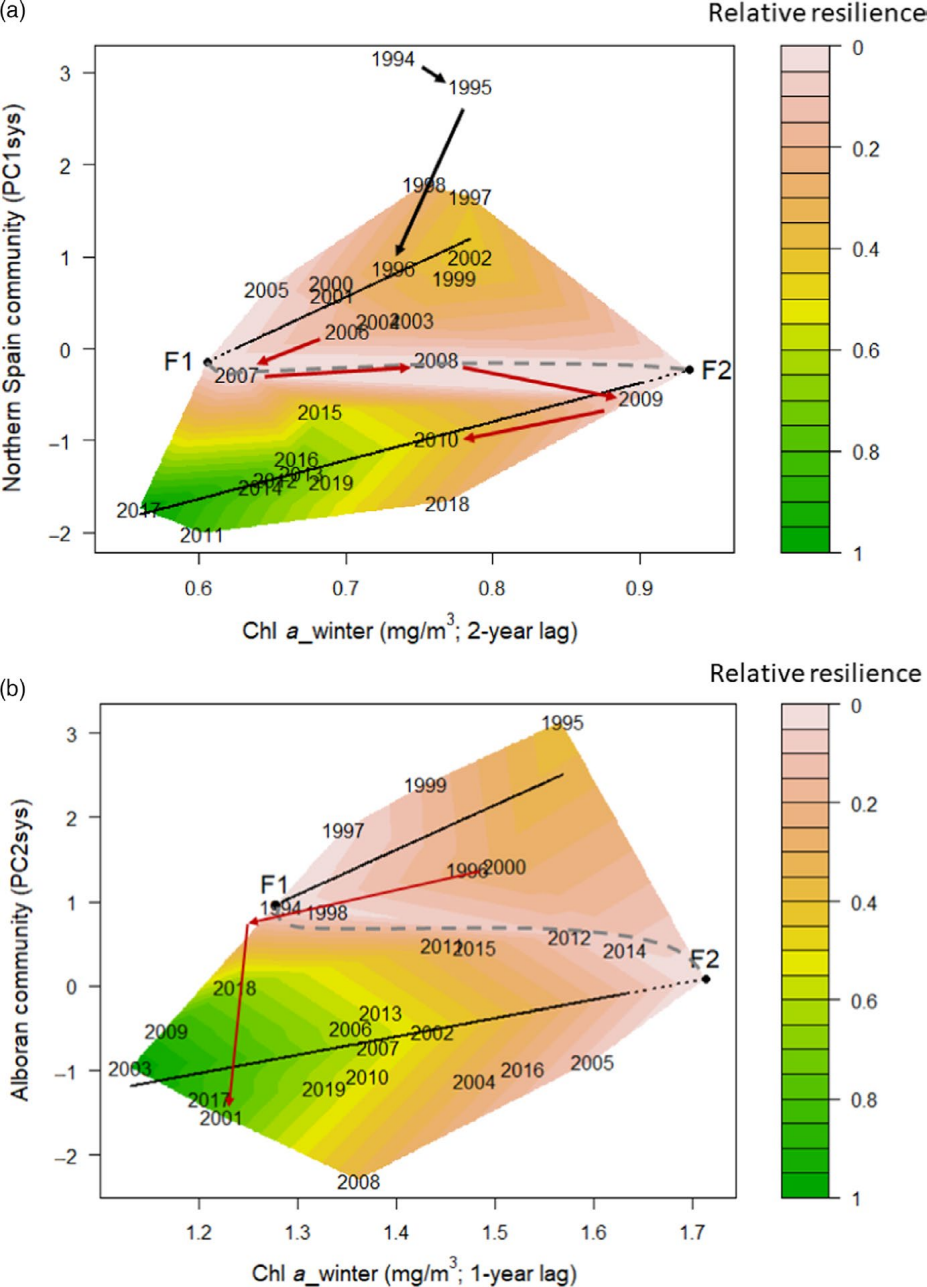
Stability landscapes and estimated relative resilience for the ecosystems of Northern Spain (a) and Alboran (b). The folded stability landscape of each ecosystem exhibited two basins of attraction and two tipping points (F1, F2). Continuous black lines indicate the attractors, dotted black lines indicate the possible extensions of the attractors, grey dashed lines indicate the boundaries of the basins of attraction, black and red arrows indicate the pathways of the regime shifts. Note that in (a) no resilience estimation was carried out for 1994, 1995, as they appear to be the ‘tail’ of an older state, and for 2008 due to its corresponding attractor being unclear

Both systems responded in a similar way to the interannual variability of chlorophyll concentration (Figure S7). In Northern Spain, the demersal community followed two consecutive periods of positive linear relationship against winter Chl *a* with a 2‐year lag (Figure 7a; Figure S6a). The community responded linearly to the decrease of Chl *a* until the late 2000s, when a local minimum of Chl *a* brought the system to a very unstable area in 2007. Subsequently, the system moved towards a new basin of attraction despite a brief increase in Chl *a* (Figure 7a). After the shift of the late 2000s, a new positive linear relationship between the system and Chl *a* was established within a new basin of attraction. With regards to resilience dynamics, the resilience of the original state gradually eroded following the change in Chl *a* towards the second half of the 2000s, and the system eventually shifted into a new state. After the shift, the system exhibited substantial hysteresis, retaining its new state even after Chl *a* values returned to their older levels during part of the 2010s. Years 1994 and 1995 appear to be the ‘tail’ of an older state, as their PC1 values differ substantially from subsequent ones (Figure 7a).

In Alboran, the system trajectory was very similar to that of Northern Spain. Nevertheless, the TGAM fit was weaker and the slope of the TGAM branches gentler. The Alboran community followed two consecutive periods of positive linear relationship against winter Chl *a* with a 1‐year lag (Figure 7b; Figure S6b). In 2001, a pronounced dip in Chl *a* brought the system to a new basin of attraction, after which, a new positive linear relationship between the system and Chl *a* was established (Figure 7b). After the shift, the system exhibited substantial hysteresis, retaining its new state despite being exposed to Chl *a* values similar to those of the 1990s during most years of the 2000s and 2010s (Figure 7a).

In both Norther Spain and Alboran, the current system states appear to be more resilient at lower concentrations of Chl *a* compared to the states pre‐shift.

## 4 DISCUSSION

The quantification of the dynamics of ecological resilience in two complex and contrasting communities of the Western Mediterranean Sea elucidated unknown ecosystem mechanisms associated with recent shifts. Intriguingly, the ecosystems investigated, while very complex and exposed to a variety of stressors, were both found to be largely driven by fluctuations in oceanic productivity with a temporal lag of 1–2 years. While different patterns were generally observed across taxonomic and functional groups in the two ecosystems, certain consistencies also emerged. Invertebrate species of short life cycle were generally favoured in recent chlorophyll‐*a* concentration regimes, particularly benthic species in the Alboran. By contrast, opportunistic fish species were relatively unfavoured in the two ecosystems in the recent periods, with an increase in commercial species of short life cycle in the Alboran and equilibrium and periodic species in the Northern Spain. Beyond the productivity dynamics driving demersal community in the two systems, our results also suggest the subtle and long‐term effects of sea warming and relative decrease in fishing. Notably, the analysis of survey data allowed a more integrated representation of both resilience and ecosystem dynamics compared to previous IRAs applied to commercial catches (Damalas et al., 2021; Ma et al., 2021; Tsimara et al., 2021; Vasilakopoulos et al., 2017).

Our results suggest that the coupling between the phenology and favourable environmental conditions (e.g. primary production blooms) is critical at the community level. While the two areas studied here are associated with different productivity regimes with a higher spatial complexity of the phytoplankton blooms in Alboran (D’Ortenzio & Ribera d’Alcalà, 2009), they exhibit consistent system responses and resilience dynamics. The lagged effects of chlorophyll‐*a* identified by the best models suggest that community impacts are driven by bottom‐up effects on the early life stages, juveniles and low trophic levels. For these groups, food web interactions and ecosystem feedbacks appear to take an average of 1–2 years to cascade through the community structure until they are sampled by the scientific survey. This lag effect is consistent with recent studies focused on the effect of sea warming on fisheries landings and their trait composition in the whole Mediterranean (Tsimara et al., 2021; Vasilakopoulos et al., 2017). The linear influence of chlorophyll‐*a* concentration on the ecosystems’ state was persistent in all periods, but the relevant relationship slopes were more gentle in the most recent period, suggesting two non‐exclusive causes: a change in the relative contribution of the different drivers affecting primary productivity in this recent period (Macias et al., 2018) and increasing importance of other hidden drivers impacting ecosystems state and resilience dynamics (e.g. Sguotti et al., 2019). Indeed, the hydroclimatic variability and the chlorophyll‐*a* variability are highly correlated in Northern Spain during the post‐shift period (Figure S8), suggesting either an increasing role of hydroclimatic conditions on the primary production dynamics or a decreasing role of the Ebro river runoff. This agrees with the known cross‐scale interactions triggering ecosystems regime shifts, in which fine‐scale processes can influence and cascade over a broad spatial extent (Selkoe et al., 2015).

Beyond consistent short‐term responses to primary productivity, other long‐term drivers likely affect systems synergistically, increasing its sensitivity to environmental variability. Sea warming is the most evident long‐term stressor, causing an increase in the biomass of species with higher temperature affinities and/or reduction of those with lower affinity (e.g. Vasilakopoulos et al., 2017). In our studied ecosystems, the community‐weighted mean temperature indicated a similar increasing pattern for the fish community, but this trend was only mirrored by invertebrates (crustaceans and cephalopods) in Northern Spain. Crustaceans and fish species of low commercial interest and high thermal affinities decreased after early 2000s in Alboran, along with the increase in some species of low thermal affinities. This is likely due to high turnover and replacement of species fostered after the early 2000s in the Alboran Sea that often receives the species runoff from the Atlantic side of the Iberian Peninsula (Real et al., 2021). Additionally, the impact of fishing has been suggested as a key chronic stressor in the Mediterranean increasing the ecosystems’ sensitivity to productivity changes (Damalas et al., 2021; Fu et al., 2018). However, a recent decrease in fishing effort and displacement of fishing activity to deeper strata in the Western Mediterranean allowed a relative recovery of highly vulnerable species in the shelf such as elasmobranchs (Ramírez‐Amaro et al., 2020) and benefitted regional fish diversity (Farriols et al., 2019). This is consistent with our results in Northern Spain and the increase in the relative contribution of some periodic commercial and equilibrium species in recent years, and also with the recent increase in commercial species of short life cycle in the Alboran Sea.

The concurrent long‐term warming, and moderate and heterogeneous decrease in fishing impact could be favouring species of short life span in recent chlorophyll‐*a* regimes. This seems to be the case in Northern Spain, where the long‐term increase in the relative contribution of species with higher thermal affinity seems to be widespread, as expected in the Mediterranean Sea and particularly for generalist groups (Doubleday et al., 2016; Moullec et al., 2019). With the change in the demersal communities’ regimes, species of short life span, such as some benthic cephalopods and crustaceans, were favoured in Northern Spain and particularly in Alboran suggesting a fuelling of the benthopelagic coupling with an increased efficiency of energy transfer to the benthic ecosystems. By contrast, opportunistic fish species were not equally favoured. This could be due to the high fish diversity in the Mediterranean, particularly for species with opportunistic life‐history strategy, which represent a high proportion in the Mediterranean fish communities (Pecuchet et al., 2017). These aspects could be of more important in systems of highly hydrodynamic and of biogeographical complexity such as the Alboran Sea (Real et al., 2021). Opportunistic species are likely to suffer from high replacement and turnover rates, as they tend to have specialized behaviour and trophic interactions and could be easily replaced under the combination of warming and changes in the productivity regime. The Alboran Sea also has an additional element of complexity due to its high hydrodynamics and low environmental predictability to which resident species and community assemblages have adapted over their evolutionary history (Winemiller & Rose, 1992), with opportunistic species becoming more sensitive and replaceable under fluctuations of primary productivity. This is consistent with the fact that the first axis of the PCA in the Alboran showed no directional trend, suggesting an efficient niche replacement and reduced impact of species coexistence.

Emerging ecosystem shifts such as the productivity‐driven shifts reported here or shifts driven by sporadic strong perturbations (extreme events or heatwaves) can be facilitated by a long‐term continuous anthropogenic degradation, warming or other major ecosystem reorganization. This can be the case in the long‐lasting multi‐impacted Mediterranean Sea where a major reorganization in species composition occurred in the mid‐1990s (also discernible here in Northern Spain), attributed to the combination of sea warming and changes in major hydroclimatic modes (Alheit et al., 2019; Vasilakopoulos et al., 2017). An accompanying transition in the life‐history traits composition has also been detected, switching traits systems to shorter life span, smaller maximum size, higher optimal temperature and shallower optimal depth (Tsimara et al., 2021). This new traits configuration could be making communities more sensitive to regional changes in primary productivity.

The regime shifts described in this study comply with the properties defined by the theory of critical transitions in complex ecological systems (Scheffer et al., 2001; Selkoe et al., 2015) and resemble other climate and/or fishing‐driven transitions displaying strong hysteresis (Litzow & Hunsicker, 2016; Möllmann et al., 2015). Nevertheless, seldom such shifts have been associated with productivity fluctuations as in our case. The presence of hysteresis implies that a new ecosystem state remains resilient and fails to reverse to its previous state even after a perturbation is reversed (Beisner et al., 2003; Scheffer et al., 2001). This kind of system dynamics implies a difference in the set of variables controlling the ecosystem response in the alternate states, with a potential change of the main environmental forcing on primary production (Figure S8). Positive feedbacks such as changes in the trophic relationships could contribute to amplify the amount of change in the system.

Most marine stewardships worldwide are currently working towards climate resiliency in ecosystems management (e.g. Holsman et al., 2019). To do that, a general systematic approach for improving resource management by learning from management outcomes (i.e. adaptive management) has become the means towards resilient socioecological landscapes (Walker et al., 2004). Management approaches which do not properly integrate across spatiotemporal scales are maladapted to unidirectional change and extreme events (Holsman et al., 2019). Our results are in this sense paradigmatic as they reveal region‐specific resilience dynamics to short‐term unexpected events and also the long‐term effects of sea warming and fishing decrease. However, further research is needed to operationalize the results obtained in this study. First, early warning indicators specific for productivity‐driven regime shifts need to be developed (Litzow & Hunsicker, 2016). Second, these indicators should be combined with the identification of the specific areas in which fine‐scale processes affecting productivity can cascade over a broad spatial and/or temporal extent (Selkoe et al., 2015). Moreover, the productivity thresholds for regime shifts identified here could be compared with forecasts of productivity in the studied areas to facilitate resilient socioecological landscapes under adaptive management frameworks. In other words, future regime shifts could be anticipated by managers to help them adapt their policies accordingly, combining strategies to manage both long‐term warming impacts and short‐term events such as productivity fluctuations and shocks.

## CONFLICT OF INTEREST

The authors have no conflict of interest to declare.

## AUTHORS' CONTRIBUTIONS

M.H. and P.V. designed the study; A.E. and C.G.‐R. collected the field biological data; E.G.G. extracted the chlorophyll data; P.V. and M.H. analysed the data, prepared the figures and lead the writing. All authors contributed substantially to the concepts and revising of the manuscript and approved the final version.

## Supporting information

Supplementary Material

## Data Availability

PCA axes and chlorophyll‐*a* data that support IRA analyses are available at https://doi.org/10.5281/zenodo.5750293 (Hidalgo, 2021).
