# Supplementary material for: Resilience dynamics and productivity‐driven shifts in the marine communities of the Western Mediterranean Sea
Source: J Anim Ecol. 2021 Dec 14;91(2):470–83. doi: 10.1111/1365-2656.13648 (PMC9300018; doi:10.1111/1365-2656.13648)
Supplement: Supplementary file 1 — Supplementary Material [file JANE-91-470-s001.docx]

**Supporting Information for**

**Resilience dynamics and productivity-driven shifts in the marine communities of the western Mediterranean Sea**

Manuel Hidalgo, Paraskevas Vasilakopoulos, Cristina García-Ruiz, Antonio Esteban, Lucía López-López, Elisa García-Gorriz

Correspondence

Manuel Hidalgo, Paraskevas Vasilakopoulos

[jm.hidalgo@ieo.es](mailto:jm.hidalgo@ieo.es); paris.vasilakopoulos@ec.europa.eu

This file includes:

Tables S1 to S2

Figures S1 and S8

Table S1. PC1 loadings (using a cut-off value of 0.4), scores for the three life-history strategies — opportunistic, periodic and equilibrium— obtained from Pecuchet et al (2016), optimal temperature and qualitative information on commercial interest for species of the Northern Spain community.

| **LOSERS** - Positive PC1 loadings (decreasing trends) | | | | | | | | |  |  | | |  | | | |  | |  | | |  | | |
| --- | --- | --- | --- | --- | --- | --- | --- | --- | --- | --- | --- | --- | --- | --- | --- | --- | --- | --- | --- | --- | --- | --- | --- | --- |
| **Class** | **Order - Family** | | **Species** | | | **PCA loadings** | **Equilibrium score** | | | | **Opportunistic score** | | | **Periodic score** | | **Optimal temperature** | | | | **Commercial** | | |  |  |
| Actinopterygii | Perciformes / Cepolidae | | *Cepola macrophthalma* | | | 0.58 | 0 | | | | 0.55 | | | 0.45 | | 16.83 | | | | 0 | | |  |  |
| Actinopterygii | Perciformes / Gobiidae | | *Lesueurigobius friesii* | | | 0.56 | 0.2 | | | | 0.8 | | | 0 | | 14.23 | | | | 0 | | |  |  |
| Actinopterygii | Perciformes / Gobiidae | | *Deltentosteus quadrimaculatus* | | | 0.51 | 0.14 | | | | 0.86 | | | 0 | | 19.36 | | | | 0 | | |  |  |
| Actinopterygii | Anguilliformes / Ophichthidae | | *Ophichthus rufus* | | | 0.50 | 0 | | | | 0.46 | | | 0.54 | | 19.24 | | | | 0 | | |  |  |
| Malacostraca | Brachyura / Polybiidae | | *Liocarcinus depurator* | | | 0.49 | - | | | | - | | | - | | 14.23 | | | | 0 | | |  |  |
| Malacostraca | Brachyura / Dorippidae | | *Medorippe lanata* | | | 0.47 | - | | | | - | | | - | | 21.04 | | | | 0 | | |  |  |
| Actinopterygii | Perciformes / Trichiuridae | | *Lepidopus caudatus* | | | 0.46 | 0 | | | | 0.34 | | | 0.66 | | 16.81 | | | | 1 | | |  |  |
| Cephalopoda | Myopsida / Loliginidae | | *Alloteuthis media* | | | 0.45 | - | | | | - | | | - | | 16.44 | | | | 1 | | |  |  |
| Actinopterygii | Perciformes / Gobiidae | | *Gobius niger* | | | 0.43 | 0.2 | | | | 0.64 | | | 0.16 | | 14.06 | | | | 0 | | |  |  |
| Actinopterygii | Pleuronectiformes / Cynoglossidae | | *Symphurus nigrescens* | | | 0.43 | 0 | | | | 0.55 | | | 0.45 | | 20.37 | | | | 0 | | |  |  |
| Actinopterygii | Perciformes / Serranidae | | *Serranus hepatus* | | | 0.42 | 0 | | | | 0.65 | | | 0.35 | | 19.12 | | | | 1 | | |  |  |
| Actinopterygii | Pleuronectiformes / Bothidae | | *Arnoglossus rueppelii* | | | 0.42 | 0 | | | | 0.63 | | | 0.37 | | 19.24 | | | | 1 | | |  |  |
| Malacostraca | Brachyura / Calappidae | | *Calappa granulata* | | | 0.41 | - | | | | - | | | - | | - | | | | 0 | | |  |  |
|  |  | | |  | | | | |  |  | | |  | | | |  | |  | | |  | | |
| **WINNERS** - Negative PC1 loadings (increasing trends) | | | | | | | | |  |  | | |  | | | |  | |  | | |  | | |
| **Class** | **Order - Family** | **Species** | | | **PCA loadings** | | | **Equilibrium score** | | | | **Opportunistic score** | | | **Periodic score** | | | **Optimal temperature** | | | **Commercial** | | |  |
| Actinopterygii | Perciformes - Sparidae | *Pagellus erythrinus* | | | -0.63 | | | 0 | | | | 0.49 | | | 0.51 | | | 18.33 | | | 1 | | |  |
| Malacostraca | Brachyura - Inachidae | *Inachus dorsettensis* | | | -0.53 | | | - | | | | - | | | - | | | 13.16 | | | 0 | | |  |
| Cephalopoda | Oegopsida - Ommastrephidae | *Illex coindetii* | | | -0.51 | | | - | | | | - | | | - | | | 20.30 | | | 1 | | |  |
| Actinopterygii | Scorpaeniformes - Peristediidae | *Peristedion cataphractum* | | | -0.50 | | | 0 | | | | 0.48 | | | 0.52 | | | 20.49 | | | 1 | | |  |
| Actinopterygii | Gadiformes - Phycidae | *Phycis blennoides* | | | -0.49 | | | 0 | | | | 0.35 | | | 0.65 | | | 14.37 | | | 1 | | |  |
| Cephalopoda | Octopoda - Octopodiidae | *Octopus vulgaris* | | | -0.49 | | | - | | | | - | | | - | | | 23.77 | | | 1 | | |  |
| Cephalopoda | Sepiida - Sepiolidae | *Sepietta oweniana* | | | -0.47 | | | - | | | | - | | | - | | | 14.47 | | | 0 | | |  |
| Actinopterygii | Pleuronectiformes - Scophthalmidae | *Lepidorhombus boscii* | | | -0.47 | | | 0 | | | | 0.5 | | | 0.5 | | | 17.10 | | | 1 | | |  |
| Actinopterygii | Perciformes - Mullidae | *Mullus barbatus* | | | -0.47 | | | 0 | | | | 0.63 | | | 0.37 | | | 19.01 | | | 1 | | |  |
| Chondrichthyes | Carcharhiniformes - Scyliorhinidae | *Scyliorhinus canicula* | | | -0.47 | | | 0.59 | | | | 0.2 | | | 0.22 | | | 14.56 | | | 1 | | |  |
| Malacostraca | Decapoda - Inachidae | *Macropodia longipes* | | | -0.46 | | | - | | | | - | | | - | | | 19.24 | | | 0 | | |  |
| Actinopterygii | Clupeiformes - Engraulidae | *Engraulis encrasicolus* | | | -0.46 | | | 0 | | | | 0.87 | | | 0.13 | | | 15.78 | | | 1 | | |  |
| Actinopterygii | Sebastidae - Scorpaeniformes | *Helicolenus dactylopterus* | | | -0.45 | | | 0.32 | | | | 0.41 | | | 0.26 | | | 19.86 | | | 1 | | |  |
| Actinopterygii | Gadiformes - Gadidae | *Trisopterus minutus* | | | -0.42 | | | 0.04 | | | | 0.49 | | | 0.48 | | | 10.99 | | | 1 | | |  |
| Actinopterygii | Perciformes - Centracanthidae | *Spicara maena* | | | -0.41 | | | 0.03 | | | | 0.52 | | | 0.45 | | | 18.90 | | | 0 | | |  |

Table S2. PC2 loadings (using a cut-off value of 0.4), scores for the three life-history strategies — opportunistic, periodic and equilibrium— obtained from Pecuchet et al (2016), optimal temperature and qualitative information on commercial interest for species of the Alboran Sea community.

| **LOSERS** - Positive PC2 loadings (decreasing trends) | | |  |  |  |  |  |  |
| --- | --- | --- | --- | --- | --- | --- | --- | --- |
| **Class** | **Order - Family** | **Species** | **PCA loadings** | **Equilibrium score** | **Opportunistic score** | **Periodic score** | **Optimal temperature** | **Commercial** |
| Actinopterygii | Perciformes - Callionymidae | *Callionymus maculatus* | 0.52 | 0 | 0.82 | 0.18 | 11.80 | 0 |
| Actinopterygii | Pleuronectiformes - Solenidae | *Microchirus variegatus* | 0.49 | 0 | 0.61 | 0.39 | 15.25 | 1 |
| Cephalopoda | Sepiidae - Sepiolidae | *Sepia elegans* | 0.47 | - | - | - | 19.41 | 1 |
| Malacostraca | Brachyura - Polybiidae | *Liocarcinus depurator* | 0.43 | - | - | - | 14.23 | 0 |
| Actinopterygii | Pleuronectiformes - Cynoglossidae | *Symphurus nigrescens* | 0.43 | 0 | 0.55 | 0.45 | 20.37 | 0 |
|  |  |  |  |  |  |  |  |  |
|  |  |  |  |  |  |  |  |  |
| **WINNERS** - Negative PC2 loadings (increasing trends) | | |  |  |  |  |  |  |
| **Class** | **Order - Family** | **Species** | **PCA loadings** | **Equilibrium score** | **Opportunistic score** | **Periodic score** | **Optimal temperature** | **Commercial** |
| Actinopterygii | Perciformes - Sparidae | *Boops boops* | -0.56 | 0 | 0.6 | 0.40 | 19.67 | 1 |
| Actinopterygii | Perciformes - Sparidae | *Pagrus pagrus* | -0.51 | 0 | 0.34 | 0.66 | 22.66 | 1 |
| Actinopterygii | Perciformes - Sparidae | *Diplodus vulgaris* | -0.49 | 0 | 0.65 | 0.35 | 19.41 | 1 |
| Actinopterygii | Perciformes - Centracanthidae | *Spicara smaris* | -0.49 | 0.18 | 0.66 | 0.16 | 18.67 | 1 |
| Actinopterygii | Perciformes - Carangidae | *Trachurus mediterraneus* | -0.48 | 0 | 0.48 | 0.52 | 18.80 | 1 |
| Actinopterygii | Perciformes - Sparidae | *Pagellus erythrinus* | -0.47 | 0 | 0.49 | 0.51 | 18.33 | 1 |
| Actinopterygii | Perciformes - Clupeidae | *Sardina pilchardus* | -0.44 | 0 | 0.66 | 0.34 | 14.89 | 1 |
| Actinopterygii | Perciformes - Sparidae | *Pagellus acarne* | -0.40 | 0 | 0.53 | 0.47 | 18.59 | 1 |


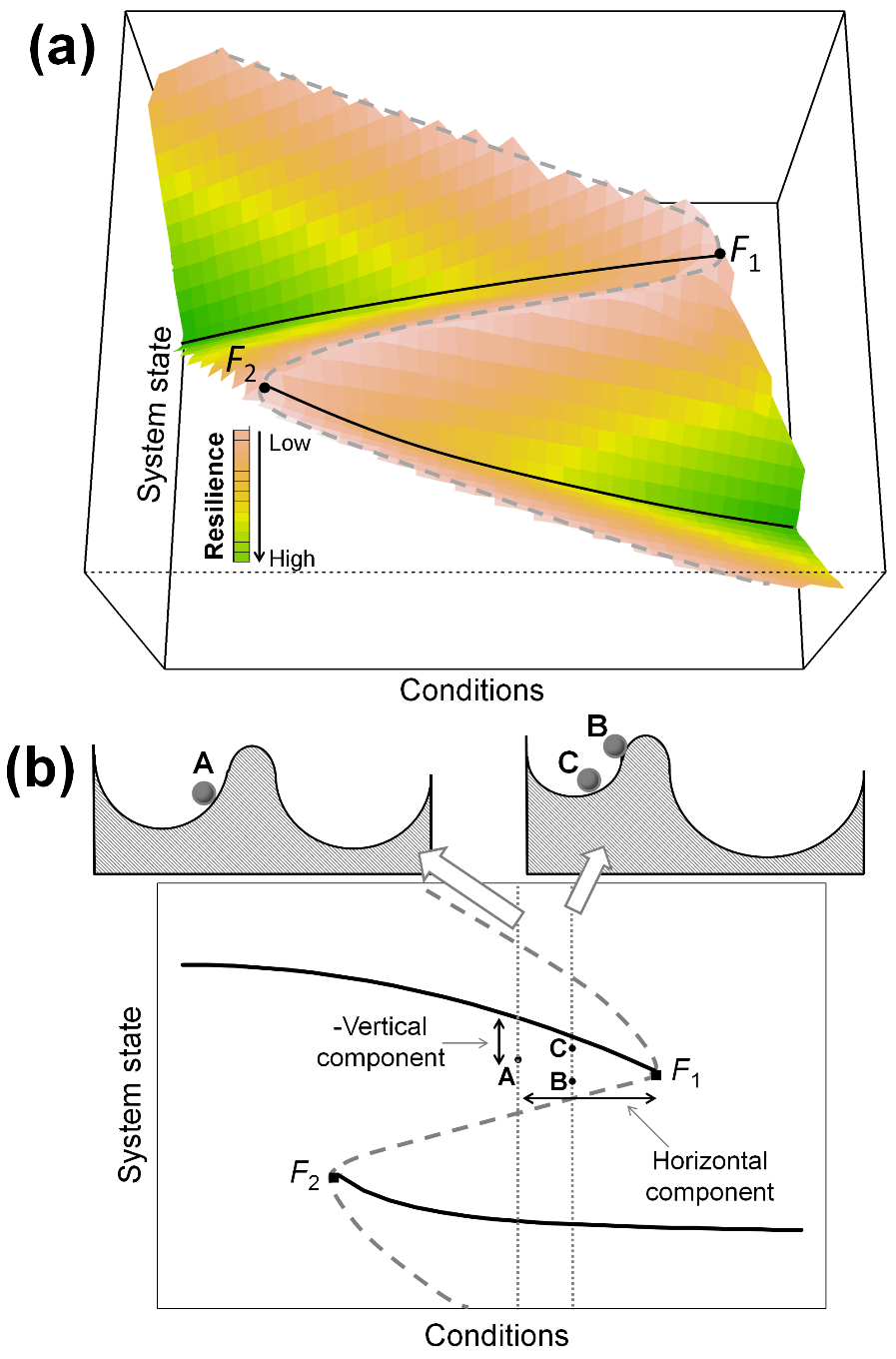


**Fig. S1** A generic folded stability landscape with two basins of attraction. System response curves (attractors) and basins’ borders are indicated by black continuous and grey dashed lines, respectively. As conditions change and the system approaches a tipping point (F1 or F2) resilience erodes and the basins of attraction become narrower and shallower (a). For the estimation of resilience in the context of the Integrated Resilience Assessment (IRA) framework, the horizontal distance of a state from the tipping point expresses its horizontal component of resilience (hComp), while the distance of a state from its attractor expresses its negative vertical component of resilience (-vComp) (b). The negative sign indicates that the greater the distance of a state from its attractor, the lower its resilience. Resilience of state A is estimated as ResA = hCompA + vCompA. States B and C have the same hComp but different vComp; hence, state C is more resilient than state B. States A and B have the same vComp but different hComp; hence, state A is more resilient than state B. Adjusted from Vasilakopoulos & Marshall 2015.


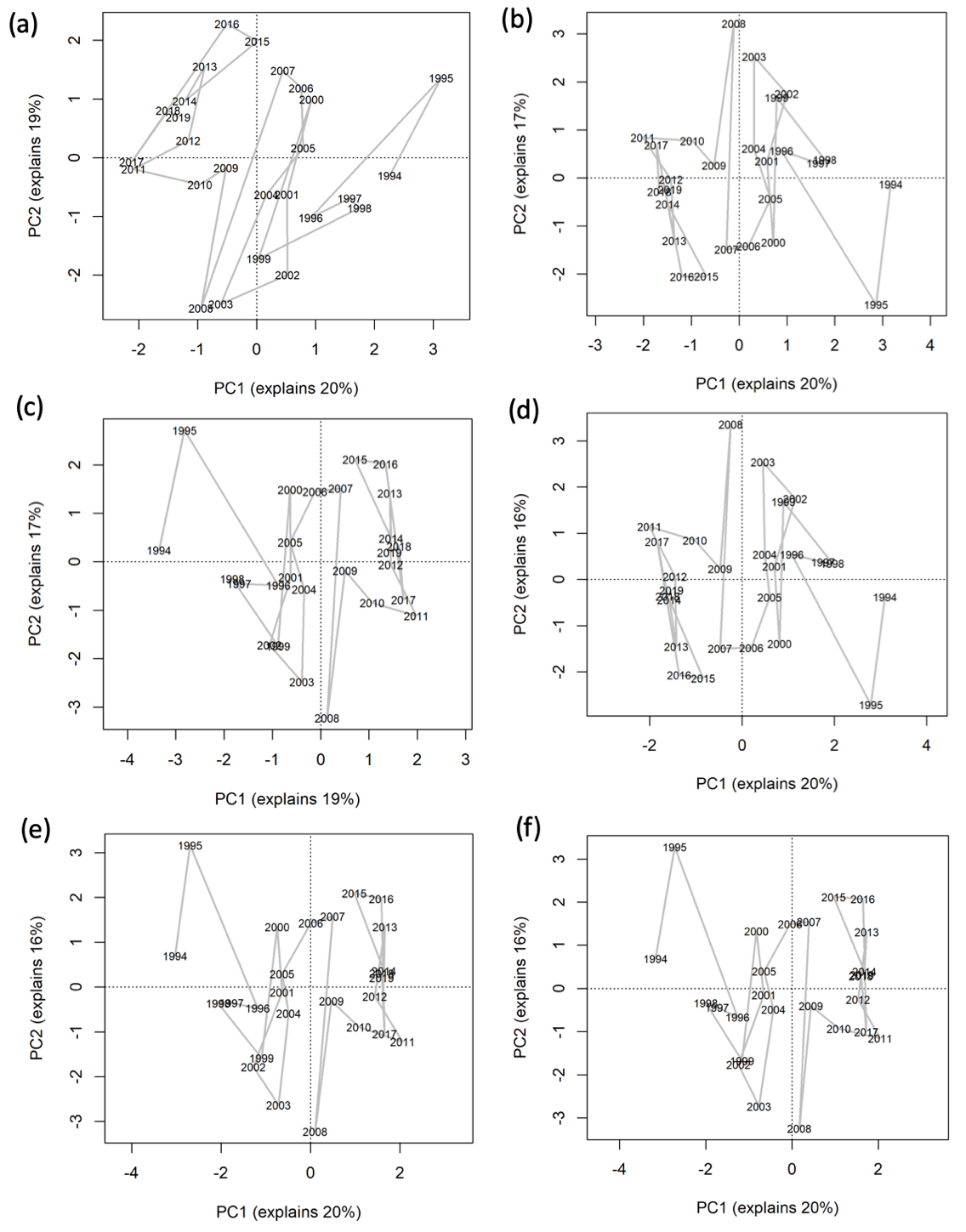


**Fig. S2.** PCA biplot for Northern Spain using a dataset including species present in 26 (a) (75 species), at least 25 (b) (97 species), at least 24 (c) (105 species), at least 23 (d) (111 species), at least 22 (e) (120 species), at least 21 years (f) (130 species).


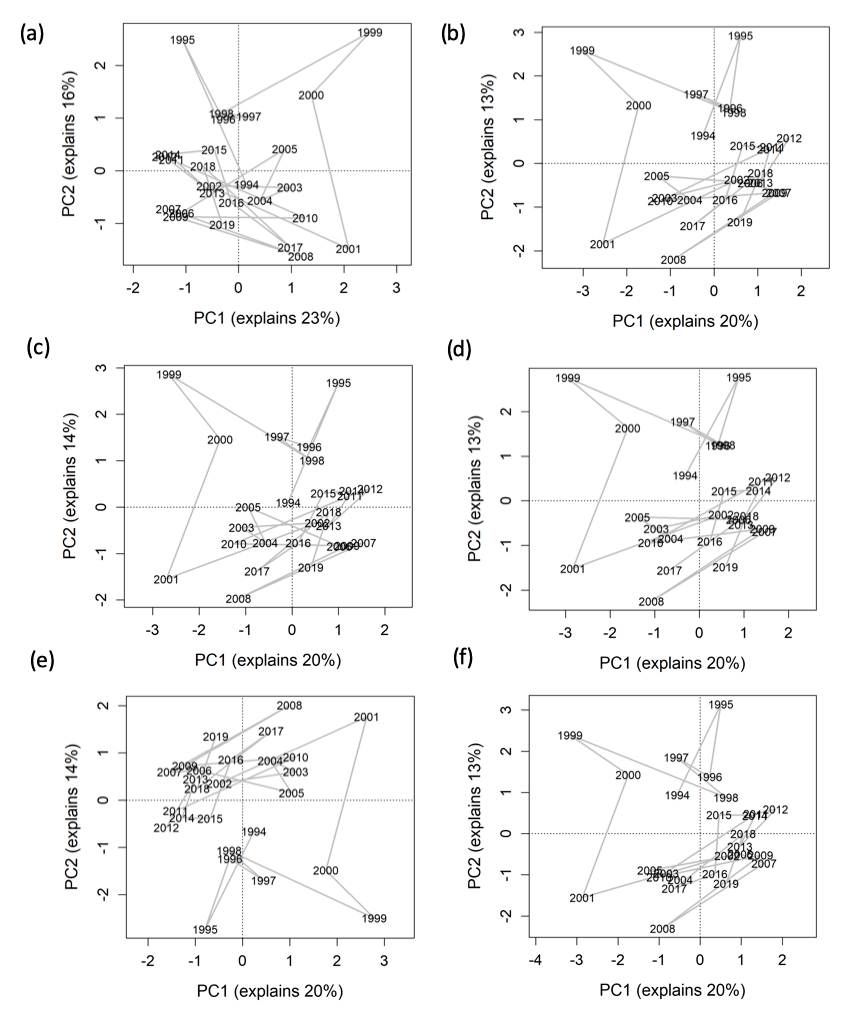


**Fig. S3.** PCA biplot for Alboran Sea using a dataset including species present in 26 (a) (38 species), at least 25 (b) (55 species), at least 24 (c) (58 species), at least 23 (d) (64 species), at least 22 (e) (69 species), at least 21 years (f) (74 species).


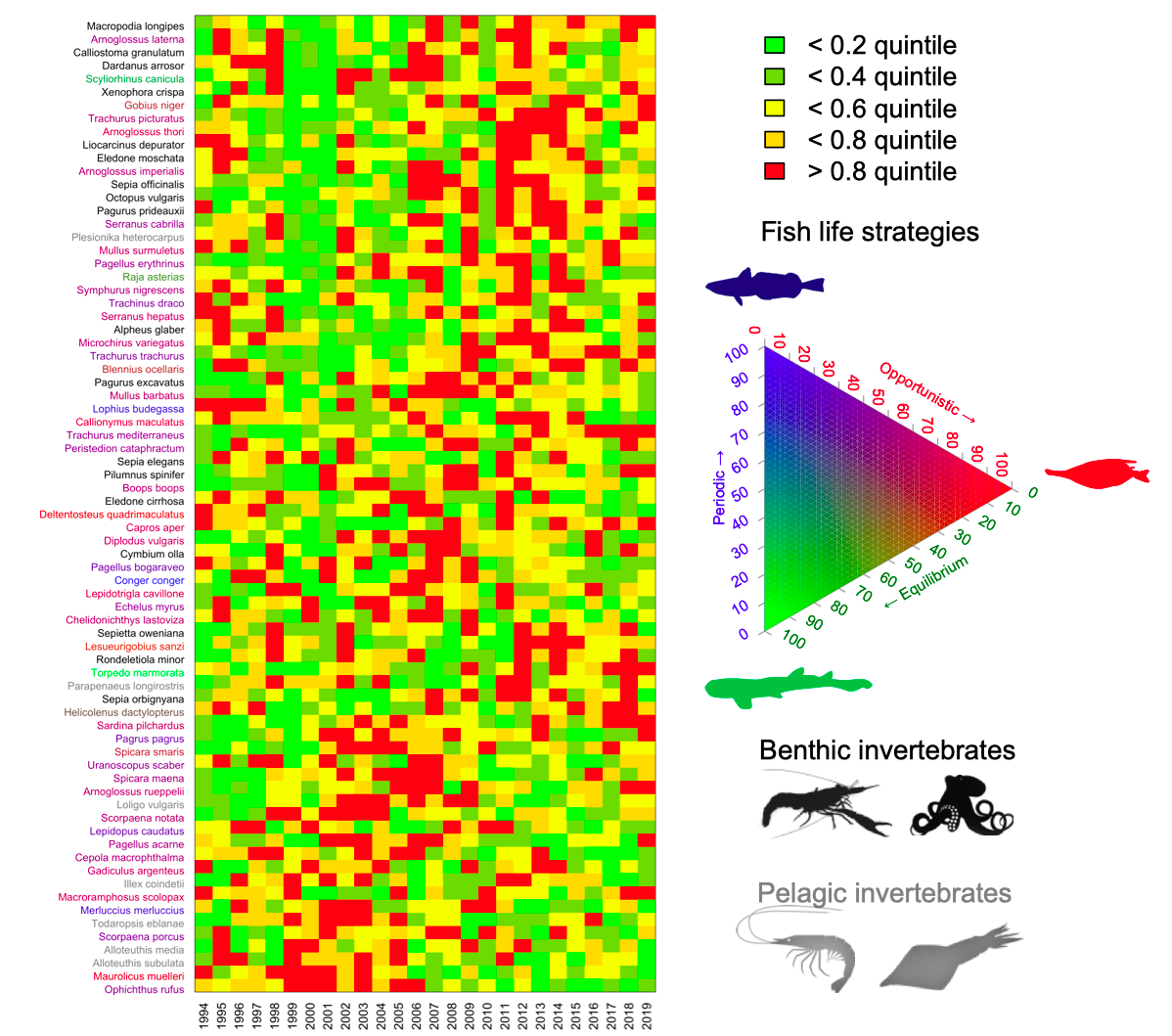


**Figure S4.** Changes in the ecosystem configuration (i.e. relative contribution of species) attending to the temporal development of standardized biomass sorted according to the loadings of PC1 of 74 species in Alboran (b).


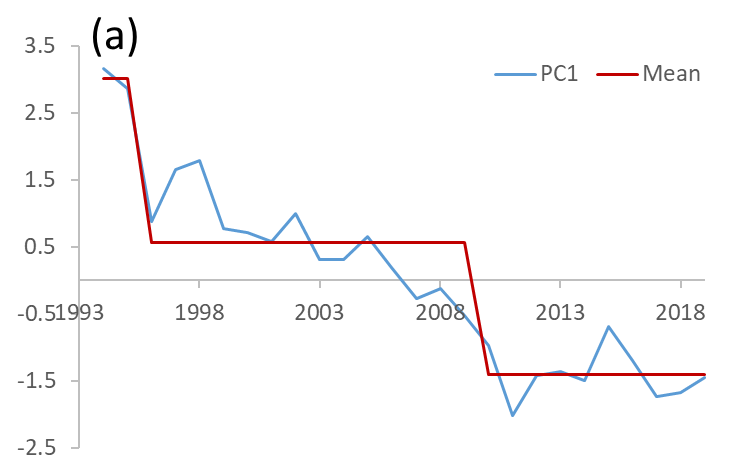


(b)


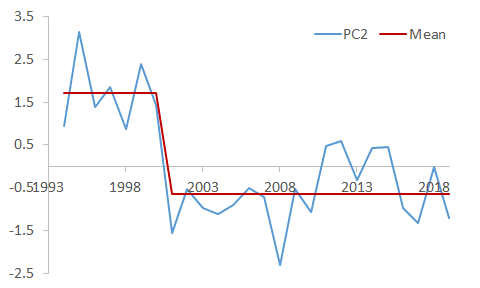


**Fig. S5.** PC1 and PC2 time-series and STARS-derived means (cut-off length: 3 years, Hubert’s parameter: 1, red noise correction: OLS, *p* = 0.05) for the Northern Spain ecosystem (a) and the Alboran Sea one (b), respectively. Two regime shifts the PC1 of Northern Spain in 1996 and 2010, and no regime shifts in PC2. One regime shift was also detected by STARS in the PC2 of Alboran in 2001, and no regime shifts in PC1.


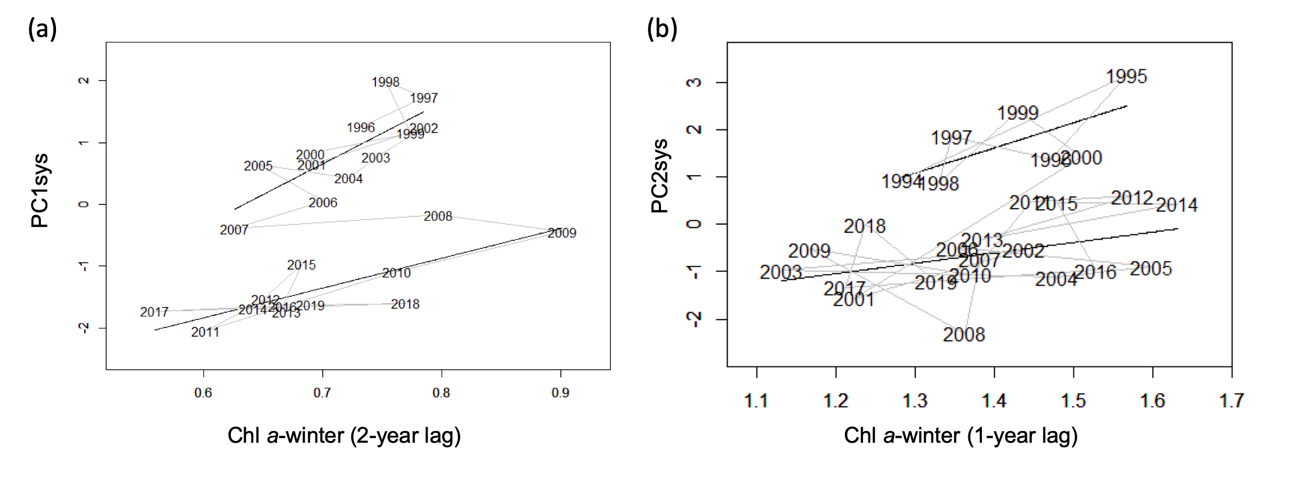


**Fig. S6**. Threshold GAM fit of the best models obtained for the Northern Spain (a) and Alboran Sea (b) showing the discontinuous response of PC1 scores used as ecosystem state indicator (PCAsys), to fluctuations of chlorophyll *a* in spring (chl *a*-spring, for the Northern Spain) and in winter (chl *a*-winter, for the Alboran Sea).

**Fig. S7.** Environment variables used for the Northern Spain (above) and the Alboran Sea (below). Regional hydroclimatic index (RHI, left, blue; details of PCA loadings in the table S3), sea surface temperature (SST, center, red) and chlorophyll *a* concertation (Chl *a*, right, green). Dashed lines indicate spring means and continuous lines winter means.


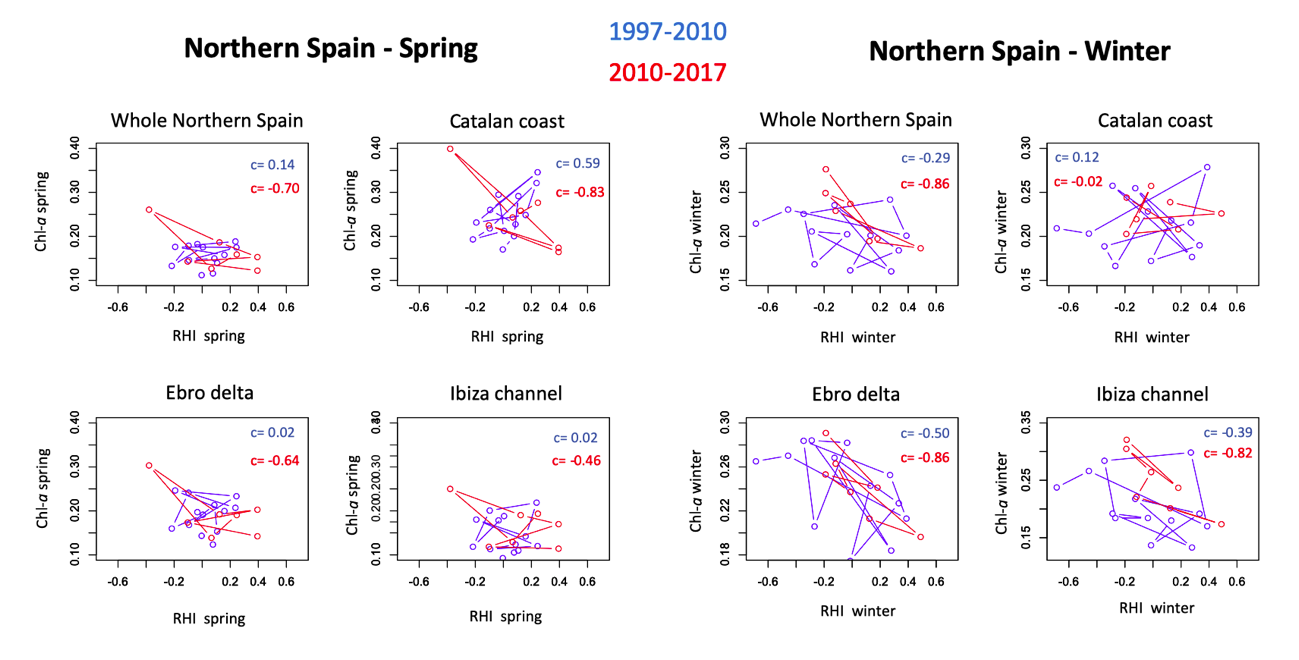


**Fig. S8.** Relationships and spearman correlation coefficients between chlorophyll-*a* and Regional Hydroclimatic Index (RHI) in the Northern Spain for spring (left ) and winter (summer), and for two periods according to the regime shift identified in the study: 1997-2010 (blue) and 2010-2017 (red). The relationships and correlation coefficients are presented for the whole region (Northern Spain), and for three sub-regions: Catalan coast (north), Ebro delta (center) and Ibiza channel (south).
